# Structural analysis of a new carotenoid-binding protein: the C-terminal domain homolog of the OCP

**DOI:** 10.1038/s41598-020-72383-y

**Published:** 2020-09-23

**Authors:** Maria Agustina Dominguez-Martin, Michal Hammel, Sayan Gupta, Sigal Lechno-Yossef, Markus Sutter, Daniel J. Rosenberg, Yan Chen, Christopher J. Petzold, Corie Y. Ralston, Tomáš Polívka, Cheryl A. Kerfeld

**Affiliations:** 1 Environmental Genomics and Systems Biology and Molecular Biophysics and Integrated Bioimaging Divisions, Lawrence Berkeley National Laboratory, Berkeley, CA 94720 USA; 2 MSU-Department of Energy Plant Research Laboratory, Michigan State University, East Lansing, MI 48824 USA; 3 Department of Biochemistry and Molecular Biology, Michigan State University, East Lansing, MI 48824 USA; 4 Institute of Physics, Faculty of Science, University of South Bohemia, Branišovská 31, 370 05 Ceske Budejovice, Czech Republic; 5 Molecular Biophysics and Integrated Bioimaging Division and Molecular Foundry, Lawrence Berkeley National Laboratory, Berkeley, CA 94720 USA; 6 Graduate Group in Biophysics, University of California, Berkeley, CA 94720 USA; 7 Biological Systems and Engineering Division, Lawrence Berkeley National Laboratory, Berkeley, CA 94720 USA; 8 Molecular Foundry, Lawrence Berkeley National Laboratory, Berkeley, CA 94720 USA

**Keywords:** Biochemistry, Biophysical chemistry, Proteins, Structural biology

## Abstract

The Orange Carotenoid Protein (OCP) is a water-soluble protein that governs photoprotection in many cyanobacteria. The 35 kDa OCP is structurally and functionally modular, consisting of an N-terminal effector domain (NTD) and a C-terminal regulatory domain (CTD); a carotenoid spans the two domains. The CTD is a member of the ubiquitous Nuclear Transport Factor-2 (NTF2) superfamily (pfam02136). With the increasing availability of cyanobacterial genomes, bioinformatic analysis has revealed the existence of a new family of proteins, homologs to the CTD, the C-terminal domain-like carotenoid proteins (CCPs). Here we purify holo-CCP2 directly from cyanobacteria and establish that it natively binds canthaxanthin (CAN). We use small-angle X-ray scattering (SAXS) to characterize the structure of this carotenoprotein in two distinct oligomeric states. A single carotenoid molecule spans the two CCPs in the dimer. Our analysis with X-ray footprinting-mass spectrometry (XFMS) identifies critical residues for carotenoid binding that likely contribute to the extreme red shift (ca. 80 nm) of the absorption maximum of the carotenoid bound by the CCP2 dimer and a further 10 nm shift in the tetramer form. These data provide the first structural description of carotenoid binding by a protein consisting of only an NTF2 domain.

## Introduction

Carotenoids are hydrophobic pigments of typically ~ 40 carbons that are widespread in nature^1^. They are most familiar in the context of photosynthesis, where they function as part of membrane protein complexes^2–4^. In contrast, very few water-soluble carotenoid-binding proteins have been described to-date. Aside from the crustacyanin protein from lobsters^5^, the most extensively characterized soluble carotenoprotein is the 35 kDa Orange Carotenoid Protein (OCP) of cyanobacteria. The OCP is a photoactive protein responsible for a non-photochemical quenching mechanism that enables cells to avoid photodamage during photosynthesis (see reviews^6,7^). The first crystal structure of the OCP revealed two discrete structural domains: an all-helical N-terminal domain (NTD) unique to cyanobacteria, and a C-terminal domain (CTD) that is a member of the widespread NTF2-like superfamily (pfam02136). A carotenoid is buried inside the protein, spanning the two domains^8^.

Increasing availability of cyanobacterial genome sequences has enabled the identification of new families of proteins that are homologous to the constituent domains of the OCP^3,8,9^. At least nine different clades of homologs to the NTD—members of the pfam09150 domain and named as Helical Carotenoid Proteins (HCPs)—can now be phylogenomically identified across diverse cyanobacteria^9^. Bioinformatic studies also revealed the presence of proteins that are homologous to the CTD of the OCP^8,9^. When recombinantly expressed in a canthaxanthin-(CAN)-producing strain of *E. coli*, the CTD binds carotenoid^10,11^ and were so named C-terminal domain-like carotenoid proteins (CCPs)^10^. Given that proteins can evolve by gene fusion it has been suggested that the full-length OCP derived from an HCP-CCP fusion event^3,7,8,10,12^.

Because HCPs and CCPs are water-soluble carotenoproteins they are valuable model systems to probe carotenoid-protein interactions and their spectral manifestations. Recently, HCPs have begun to be biochemically and structurally characterized^9,13–15^, revealing that the HCPs are monomers that bind a single carotenoid^9,14^.

In contrast, the CCPs are poorly characterized. When they were originally identified, it was suggested that CCPs interact with HCPs to form OCP-like heterodimers that function to tune photoprotection^3,8,9,12^. More recently, CCPs have been suggested to donate carotenoids to HCPs^16,17^. An apo-CCP has been structurally characterized^18,19^, but the structure of a CCP with bound carotenoid has not been determined. The CCPs are members of the ubiquitous NTF2-like superfamily, which has an α/β fold that forms a cone-like shape, with a cavity inside that acts as a molecular container adaptable for a broad range of functions^20^. There are 186 NTF2-like domain structures in the Protein Data Bank (PDB) but aside from the OCP, in which approximately half of the carotenoid is buried in the CTD, no structures containing a carotenoid are known.

The genome of the filamentous, chromatically acclimating cyanobacterium *Tolypothrix* sp. PCC 7601 (also known as *Fremyella diplosiphon* UTEX481), hereafter *Tolypothrix*) encodes two OCPs^21^, three HCPs (HCP1, HCP2, and HCP3) and one CCP that belongs to one of two groups distinguished by containing either a conserved Phenylalanine (CCP1) or a Cysteine (CCP2) residue at position 105 (Figure S1). Here, we structurally characterized *Tolypothrix* CCP2 (Cys-containing, IMG gene ID: 2501541405) with its natively bound carotenoid, CAN. Structurally, we find that the holo-CCP2 is a disulfide linked dimer, in which a single CAN spans the monomer–monomer interface and maintains the dimeric assembly even after reduction of the disulfide. Using XFMS, we identified the specific residues for carotenoid binding that partially account for the extreme red shift (80 nm) of the absorption maximum. Using SEC-SAXS-MALS we were also able to model the 3D structure of the holo-CCP2 tetramer that forms in a concentration-dependent manner and that exhibits a further spectral red-shift. Our results provide new structural insight into pigment-protein and protein interactions in this new carotenoid-binding protein family.

## Materials and methods

### Overexpression and purification of CCP2 in *Tolypothrix*

To construct an overexpression vector with CCP2 N-terminally His-tagged (10X-His), the gene was PCR-amplified from genomic DNA of *Tolypothrix* using primers SL3 (5′-TGAACCCATATGACTACTGCTGAATTTAA-3′) and SL4 (5′-TGAACCGGATCCTTAATCACGACGCATAGCGA-3′), cloned in pET16b (*Novagen*) between NdeI and BamHI and then subcloned into pRL3810^22^, downstream of the constitutive *apcA* promoter^23^. The resulting plasmid (pSL153) was introduced into *Tolypothrix *strain SF33, a shortened-filament strain^24^, by conjugation as described^25^. The CCP2 overexpression strain was grown in buffered BG-11 pH 8.0 medium supplemented with 25 μg/mL of kanamycin. The holo-CCP2 was isolated according to the method described previously^14^ with a modified lysis buffer (50 mM Tris pH 8.0, 200 mM NaCl, and 10% glycerol containing *DNase*I (*Sigma-Aldrich*), protease inhibitor cocktail (*Sigma-Aldrich*), Lysozyme (*Sigma-Aldrich*), 1 mM EDTA and 0.5 mM PMSF). 30 mg of purified CCP2 was obtained from an 18 L culture.

### Expression and purification of apo-CCP2 in *E. coli*

To produce apo-CCP2 in *E. coli*, C-terminally His-tagged (6X-His) *Tolypothrix* *CCP2* was generated by PCR using primers SL25 (5′-TCCTTAGTGATGATGGTGGTGATGATCACGACGCATAGCGA-3′) and SL26 (5′-CCATGGGCACTACTGCTGAATTTAACAA-3′), adding a His-Tag on the reverse primer, cloned in pET16b vector (*Novagen*) between NcoI and BamHI sites and expressed in BL21(DE3) cells. The purification of apo-CCP2 was carried out as previously described^14^.

### Native polyacrylamide gel electrophoresis analysis

The CCP2 samples were subjected to electrophoretic separation in a 15% polyacrylamide gel (*Bio-Rad*) and run at a voltage of 200 V at 4 ºC for 60 min. Gels were either visualized directly or were stained with Coomassie Brilliant Blue G250 for full protein visualization.

### Extraction and analysis of carotenoids by LC–MS/MS

Samples of purified CCP2 were extracted with acetone at − 20 °C for 30 min. After centrifugation (21,000 *g* at 4 °C for 10 min), the supernatant was incubated at − 20 °C for 30 min and then centrifuged. This procedure was repeated several times until a white pellet was obtained, indicating the absence of pigment in the protein sample. The carotenoid composition was analyzed as described previously using LC-DAD-MS^14^.

### Analytical size exclusion chromatography (SEC)

To estimate the oligomeric state of CCP2 holo- and apo- proteins analytical SEC was carried out as described previously^14^.

### Measurement of ultraviolet–visible (UV–Vis) spectra in solution

Samples were buffer-exchanged into 50 mM Tris–HCl (pH 8.0), and 200 mM NaCl before spectroscopic measurements. The ratio A_max peak_/A_280_ was determined in order to evaluate the purity of the holoproteins obtained. Samples were also treated with different concentrations of TCEP (Tris(2-carboxyethyl) phosphine) and DTT (1,4-Dithiothreitol) and incubated them for 30 min before spectroscopic measurements. UV–Vis absorption spectra were collected with a Cary 60 spectrophotometer (*Agilent Technologies Inc*).

### XFMS measurement, mass spectrometry and data analysis

Purified holo-CCP2 (overexpressed in *Tolypothrix)* and apo-CCP2 (overexpressed in *E. coli*) were exchanged into 10 mM potassium phosphate (pH 7.0), 100 mM NaCl by SEC on a Superdex-75 10/300 GL column before XFMS experiments. Protein samples were irradiated in the millisecond time range at beamline 3.2.1 at the Advanced Light Source (ALS, Berkeley California). All samples, including the control (no X-ray irradiation), were subjected to cysteine-alkylation and desalting before overnight trypsin and endoproteinase GluC digestions at pH 8.0 and 37 °C. Proteolyzed samples were analyzed in an Agilent 6550iFunnel Q-TOF mass spectrometer (*Agilent Technologies Inc*) coupled to an Agilent 1290 LC system (*Agilent Technologies Inc*) using Ascentis Peptides ES-C18 reverse-phase column (2.1 mm × 100 mm, 2.7-μm particle size; *Sigma-Aldrich*) as previously described^26^. The unmodified and modified peptide fragments were identified by Mascot database search of the tandem mass spectrometry data collected in the data-dependent mode. The abundance (peak area) of the identified unmodified and modified peptides at each irradiation time point were measured from their respective extracted ion chromatogram of the mass spectrometry data collected in the precursor ion mode using Agilent MassHunter V 2.2 software (*Agilent Technologies Inc*). The fraction unmodified for each peptide was calculated as the ratio of the integrated peak area of the unmodified peptide to the sum of integrated peak areas from the modified and unmodified peptides. The dose–response curves (fraction unmodified vs. X-ray exposure) were fitted to single exponential functions in Origin Version 7.5 (*OriginLabs*). The rate constant, *k* (s^−1^), was used to measure the reactivity of a side chain towards hydroxyl radical-induced modification^27^.

### Small-angle X-ray scattering and multi-angle light scattering data acquisition with in-line size-exclusion chromatography (SEC-SAXS-MALS)

For SEC-SAXS-MALS experiments, 60 μL of samples containing either 5 mg/mL of apo-CCP2 dimer, 5 mg/mL apo-CCP2 tetramer, or 5 mg/mL holo-CCP2 were prepared in 10 mM Na-phosphate (pH 7.0), 100 mM NaCl. SEC-SAXS-MALS data were collected at the ALS beamline 12.3.1^28^. X-ray wavelength was set at λ = 1.127 Å and the sample to detector distance was 2,100 mm resulting in scattering vectors, q, ranging from 0.01 to 0.4 Å^−1^. The scattering vector is defined as q = 4πsinθ/λ, where 2θ is the scattering angle. All experiments were performed at 20 °C and data was processed as described^29^. Briefly, a SAXS flow cell was directly coupled with an online Agilent 1260 Infinity HPLC system (*Agilent Technologies Inc*) using a Shodex KW802.5 column. The column was equilibrated with running buffer (10 mM Na-phosphate pH 7.0, 100 mM NaCl) with a flow rate of 0.5 mL/min. 55 µL of each sample was run through the SEC and 3 s X-ray exposures were collected continuously during a 30 min elution. The SAXS frames recorded prior to the protein elution peak were used to subtract all other frames. The subtracted frames were investigated by radius of gyration (Rg) derived by the Guinier approximation I(q) = I(0) exp(− q^2^*Rg*2/3) with the limits q*Rg < 1.5. The elution peak was mapped by comparing integral of ratios to background and Rg relative to the recorded frame using the program ScÅtter (Fig. 4A). Uniform Rg values across an elution peak represent a homogenous assembly. Final merged SAXS profiles, derived by integrating multiple frames across the elution peak, were used for further analysis including a Guinier plot which determined the aggregation free state (Figure S4). The program ScÅtter was used to compute the pair distribution function (P(r)) (Fig. 4B). The distance r, where P(r) approaches zero intensity, identifies the maximal dimension of the macromolecule (Dmax). P(r) functions were normalized based on the molecular weight of the assemblies as determined by ScÅtter using volume of correlation Vc (Supplementary Table 1). Eluent was subsequently split 3 to 1 between the SAXS line and a series of UV at 280 and 260 nm, MALS, quasi-elastic light scattering (QELS), and refractometer detector. MALS experiments were performed using an 18-angle DAWN HELEOS II light scattering detector connected in tandem to an Optilab refractive index concentration detector (*Wyatt Technology*). System normalization and calibration was performed with bovine serum albumin using a 45 μL sample at 10 mg/mL in the same SEC running buffer and a dn/dc value of 0.19. The light scattering experiments were used to perform analytical scale chromatographic separations for Mw determination of the principle peaks in the SEC analysis (Fig. 4A). UV, MALS, and differential refractive index data were analyzed using Wyatt Astra 7 software (*Wyatt Technology*) to monitor the homogeneity of the sample across the elution peak, complementary to the above-mentioned SEC-SAXS signal validation (Fig. 4A).

### SAXS solution structure modeling

Homology atomistic model including missing C- and N-terminal residues of apo-CCP2 dimer, trimer, and tetramer were built based on the crystal structure of *Anabaena* Apo-CCP2 (PDB 6FEJ)^19^ by MODELLER^30^. For the holo-CCP2 dimer, homology models for each monomer were built based on the CTD from the crystal structure of the *Arthrospira maxima* OCP (PDB 5UI2)^8^. The carotenoid, 3′-hydroxyechinenone, in the OCP structure was used as template for the SAXS based modelling of the holo-CCP2. One monomer of the CCP2 was then superimposed on the CTD of the OCP together with its bound carotenoid, and a second CCP2 monomer was added on the free end of the carotenoid, based on the same CCP2 interactions with the symmetrical half of the carotenoid originally bound by the NTD of the OCP, with manual optimization to satisfy the intra-monomer disulfide bond as well as results from the XFMS experiments. Minimal molecular dynamics (MD) simulations were performed on flexible regions in the models by the rigid body modeling strategy BILBOMD in order to optimize the conformational space of C- and N- termini^31^. The experimental SAXS profiles were then compared to theoretical scattering curves generated from atomistic models using FoXS^32,33^.

### Structure visualization and accession codes

Structural models were visualized using PyMOL (The PyMOL Molecular Graphics System, version 1.7 Schrödinger, LLC) and UCSF Chimera^34^. Structural models and SAXS profiles are deposited with the SASBDB (https://www.sasbdb.org/)^35^ using the following accession codes: apo-dimer (SASDHC6, https://www.sasbdb.org/data/SASDHC6/22b40stj8c/), apo-tetramer (SASDHE6, https://www.sasbdb.org/data/SASDHE6/p3vm5hznjw/), holo-dimer (SASDHD6, https://www.sasbdb.org/data/SASDHD6/bw0j2b9vby/) and holo-tetramer (SASDHF6, https://www.sasbdb.org/data/SASDHF6/mpq1b4a799/).

## Results

### Biochemical, spectroscopic, and pigment characterization of CCP2 from *Tolypothrix* PCC 7601

**Figure 1:**
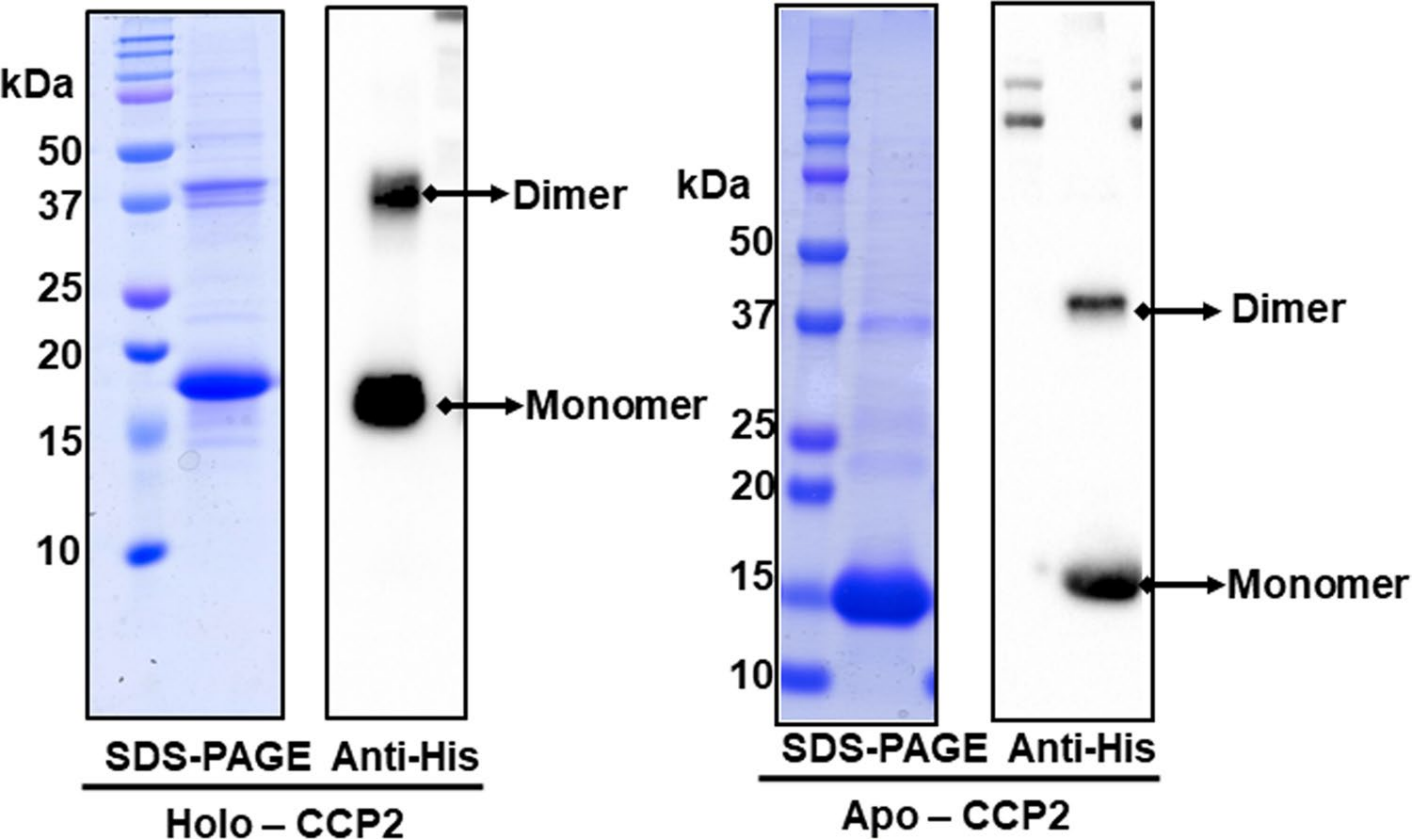
Characterization of CCP2 purified from *Tolypothrix* by denaturing gel electrophoresis. Coomassie blue-stained SDS-PAGE and anti-his immunodetection of holo-CCP2 (left) and apo-CCP2 (right).

The *Tolypothrix* CCP2 (conserved Cys at residue 105), with a calculated molecular mass of 15.65 kDa and a predicted isoelectric point of 4.77, was tagged and overexpressed in *Tolypothrix* to obtain holoprotein (hereafter holo-CCP2) binding its native carotenoid. Apo-CCP2 was produced in *E. coli*. (Fig. 1). Purity was verified by SDS-PAGE (Fig. 1). The purified holo-CCP2 was purple (Figure S7), confirming binding of the carotenoid. The LC–MS/MS analysis showed that all of the bound carotenoid is the ketocarotenoid CAN. We detected two isomers: 86.5% all-trans-CAN and 13.5% cis-CAN (Figure S2).

**Figure 2:**
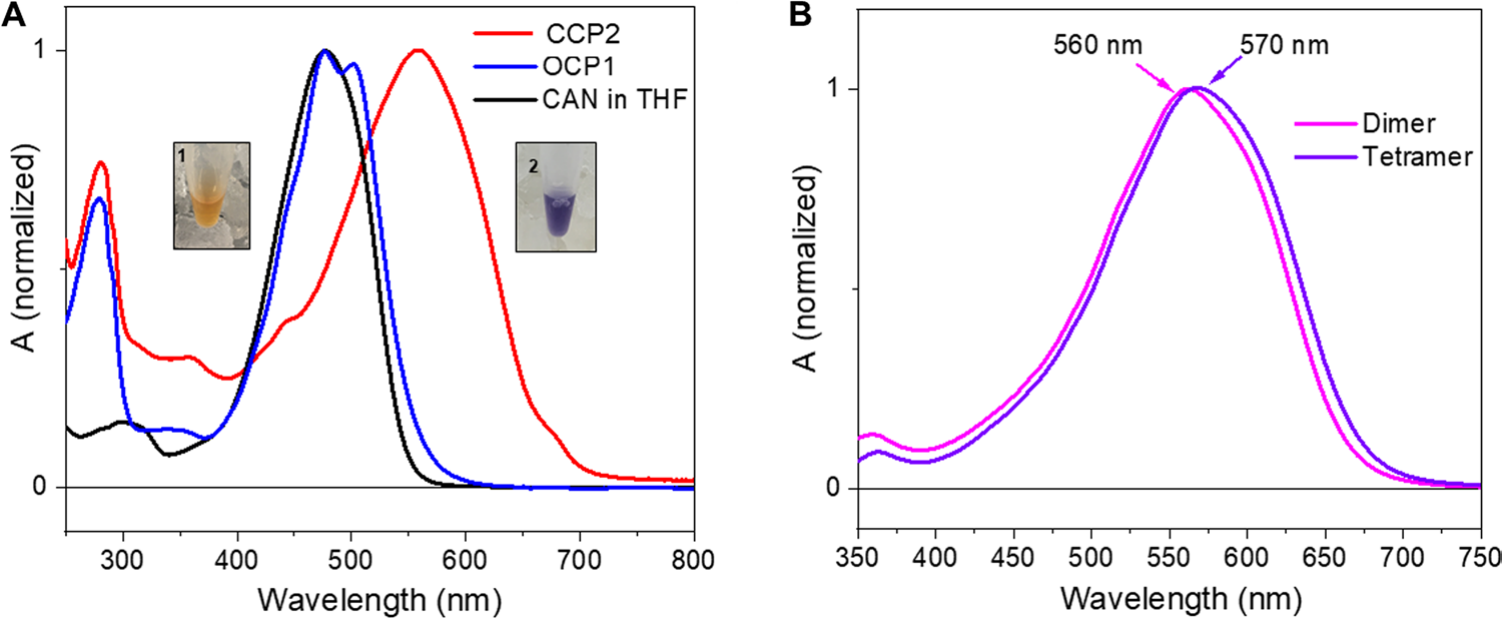
UV–Vis absorption spectra. (**A**) Absorption spectrum of CCP2 (red line), CAN-OCP1 from *Tolypothrix* (blue line, taken from^36^) and CAN in THF (black line). The color of CAN dissolved in organic solvent (1) and the purified protein in solution (2) are shown in the inset. (**B**) Absorption spectrum of CCP2 after SEC. The fractions correspond to the dimer peak (pink line) and the tetramer peak (purple line).

The UV–Vis absorption spectrum of the CCP2 shows a maximum at 560 nm with no vibrational features (Fig. 2A); the two weak shoulders at 440 and 680 nm are most likely due to chlorophyll contamination (the maxima match the expected positions of the Q_y_ (680 nm) and Soret (440 nm) bands of Chlorophyll a). When we compare the CCP2 absorption spectrum with that of CAN in solution (in tetrahydrofuran (THF)), there is an extreme red shift of the maximum by 80 nm (Fig. 2A). Such an extreme red shift is also observed when we compare CCP2 with the previously-published spectrum of *Tolypothrix* OCP1 binding CAN (Fig. 2A)^36^. However, the CAN in OCP1 exhibits a clear vibrational structure of the absorption band due to restriction of conformational flexibility^36^. No such vibrational bands are observed in the absorption spectrum of CCP2, suggestive of increased conformational freedom of CAN within CCP2 relative to what is observed in OCP1.

In SDS-PAGE under reducing conditions (established by adding β-mercaptoethanol to the loading buffer) both holo- and apo-CCP2 migrated predominantly as monomers (MW 16 kDa), although a portion of each migrated as an apparent dimer (Fig. 1). Immunoblotting against the His-Tag confirmed that both bands correspond to CCP2. Most likely the appearance of the upper, putative dimer, band is due to an incomplete reduction of the sample.

**Figure 3:**
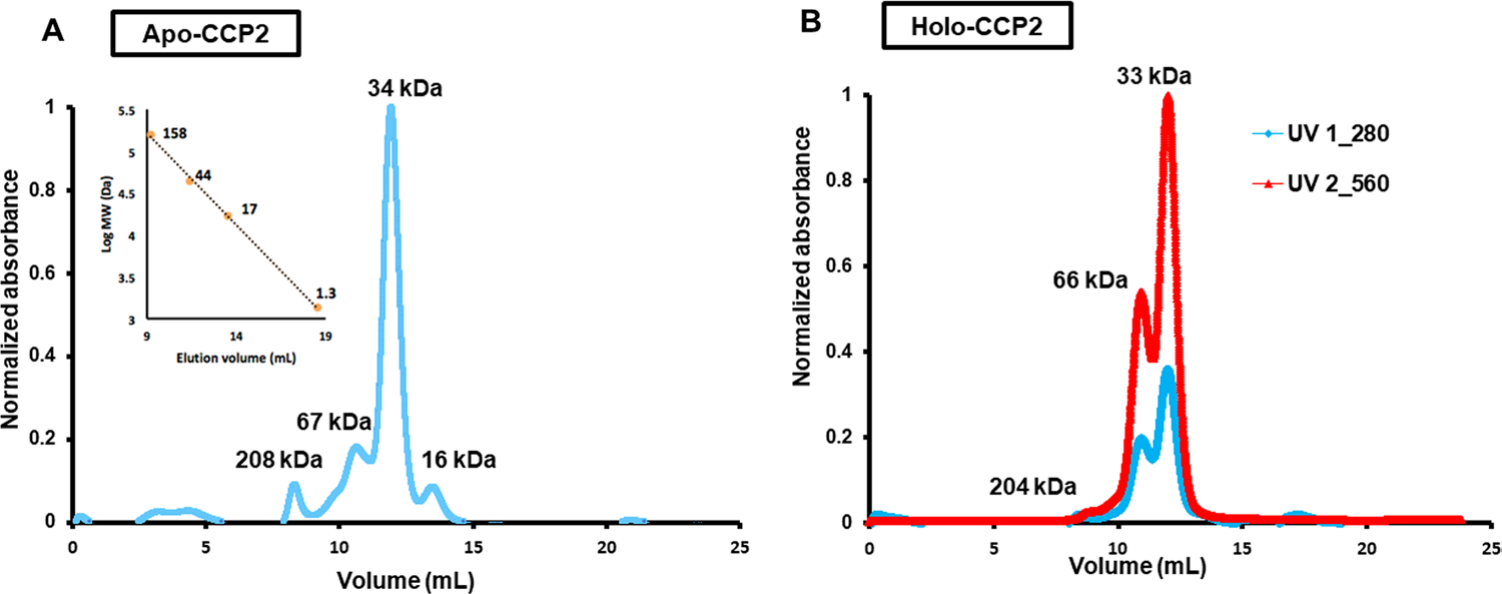
Oligomeric state of holo- and apo-CCP2. Apo- (**A**) and holo- (**B**) CCP2 oligomerization states analyzed by analytical SEC with a Superdex 75 column. Protein molecular weight standards were used to estimate the molecular mass for each species (inset graph **A**).

The native oligomerization states of holo- and apo-CCP2 were analyzed by native-PAGE and analytical SEC (Fig. 3 and Figure S7, respectively). In native-PAGE, both holo- and apo- forms migrated as a single band at approximately equivalent positions; the holo-CCP2, which retains its color and carotenoid, migrates slightly above apo-CCP2 (Figure S7), both near the 66 kDa marker. We examined the concentration dependence of migration for the holo-CCP2; it showed the same band position in a concentration range between 0.25 and 2.6 mg/mL (Figure S3A). On analytical SEC (Fig. 3), holo-CCP2 eluted as a dimer (MW 33 kDa) preceded by a small amount of tetramer (MW 66 kDa). Absorption in the visible region (~ 560 nm) for both dimer and tetramer indicates that the pigment is bound in both forms. The apo-CCP2 sample showed a SEC elution profile similar to holo-CCP2 but with an additional, colorless peak corresponding to the monomer form (MW 16 kDa). The proportion of tetramer versus dimer is higher in the holo- than the apo-form, with the dimer as the predominant species in both apo- and holo-CCP2. Interestingly, when we measured the absorption spectrum of the tetramer form, we observed a further red shift by 10 nm (Fig. 2B).

### Oligomeric state analysis of CCP2 by SEC-SAXS-MALS

**Figure 4:**
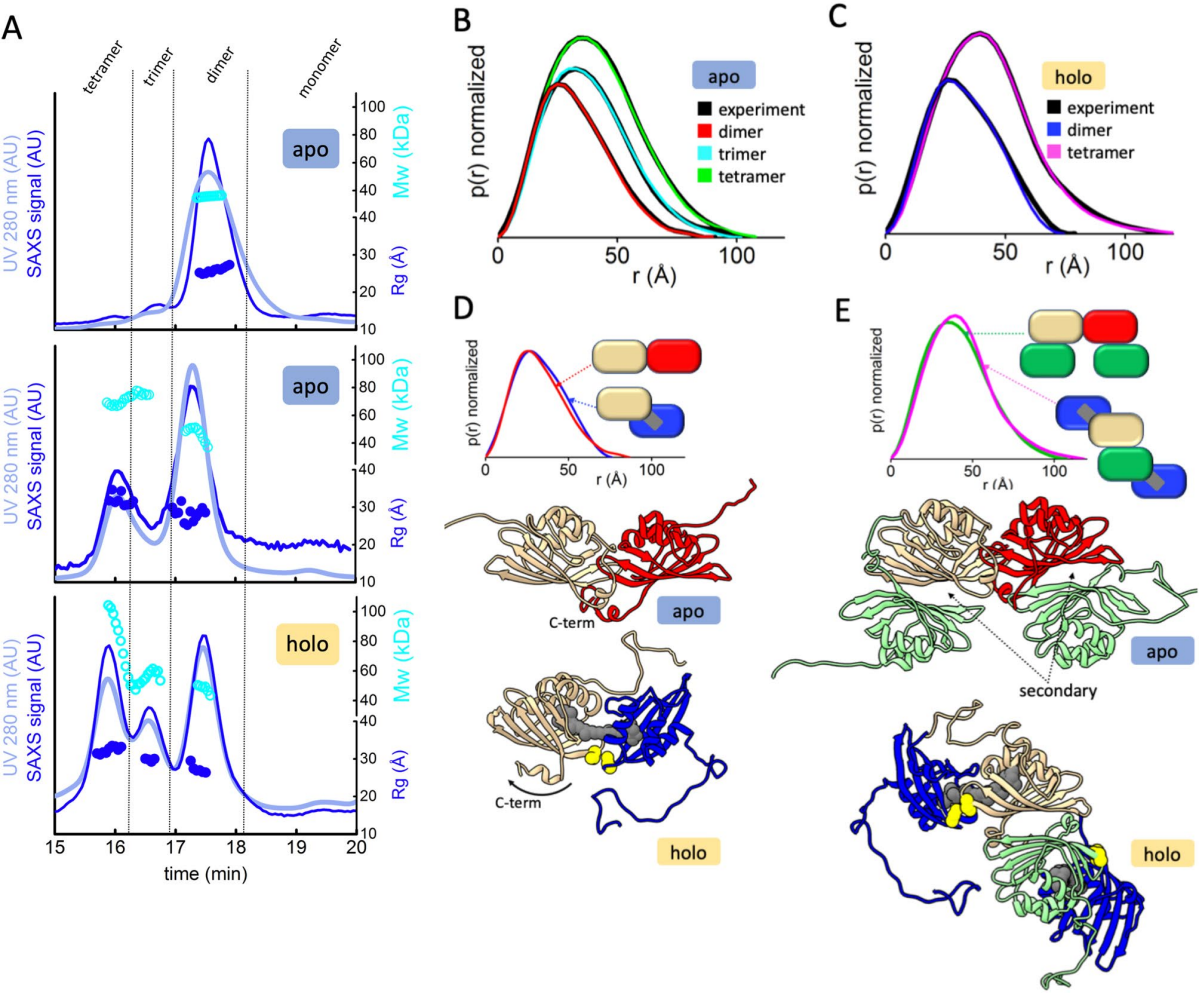
Global structural analysis by SEC-SAXS-MALS. (**A**) Top: SEC-MALS-SAXS chromatograms for apo-CCP2 dimer (5 mg/mL, apo1), apo-CCP2 tetramer (10 mg/mL, apo2), holo-CCP2 (5 mg/mL holo). Solid lines represent the UV 280 nm (light blue) or integrated SAXS signal (dark blue) in arbitrary units, while symbols represent molecular mass (cyan) and Rg values for each collected SAXS frame (dark blue) versus elution time. (**B**,**C**) p(r) functions calculated for the experimental data (black) of apo-CCP2 dimer, trimer, and, tetramer; and holo-CCP2 dimer and tetramer, shown in Figure S4. The area of the p(r) is normalized relative to the MW estimated by SAXS^48^ and is listed in Table S1. Theoretical p(r) function is calculated from the theoretical SAXS curves shown in Figure S4 of the corresponding models shown in (**D**,**E**). (**D**) Comparison of the experimental p(r) functions of holo-CCP2 dimer (blue) and apo-CCP2 dimer (red) together with the derived 3D structural models (see Materials and methods). C-terminal region (C-term) is highlighted. (**E**) Comparison of the experimental p(r) functions of holo-CCP2 tetramer (magenta) and apo-CCP2 dimer (green) together with the derived 3D structural models (see Materials and methods). The position of the conserved cysteine is highlighted in yellow.

We used SEC-SAXS-MALS to obtain the overall arrangement of apo- and holo- CCP2 in their different oligomerization states^37^. MALS and SAXS data shows the presence of apo-CCP2 dimer, trimer, and tetramer in solution (Fig. 4A,B, Table S1). In contrast, the holo-CCP2 sample shows only dimer and tetramer species in solution (Fig. 4A,C, Table S1). The presence of the tetramer correlates with increasing protein concentration (Figure S3B). Other than the definitive presence of an apo-CCP2 trimer, these results are in good agreement with the data obtained by analytical SEC (Fig. 3).

While the overall fold of the CCP is known, based on its membership in the NTF2-like superfamily, p(r) function calculated from SAXS data showed significant differences between apo- and holo- CCP2 dimer arrangement, indicating a rod-like arrangement for the apo-CCP2 dimer (Fig. 4D). The p(r) of the holo-CCP2 dimer, with its relatively bulky form and shoulder at ~ 50 Å, suggests a slight separation of two CCP2 monomers. This observation agrees with smaller Rg values of the apo-CCP2 dimer (Rg ~ 24.0 Å apo-CCP2 dimer vs. Rg ~ 25.5 Å holo-CCP2 dimer, see Table S1). In addition, a remarkable difference between the apo-CCP2 and the holo-CCP2 dimer was in the position of the C-terminal region (L128-D141, Figure S1). In the apo-form structure, the C-terminal region contains a short helix of six residues that is located at the dimer interface between the two monomers. The model for the holo-form suggests that this region opens up when the dimer contains the pigment (Fig. 4D).

To validate this conformational arrangement of the CCP2 dimer in the holo-form, we compared the apo-CCP2 dimer (from the available crystal structure, PDB 6FEJ^19^) with a model of the holo-CCP2 dimer with the carotenoid spanning the two domains (see Materials and methods). In our holo-CCP2 dimer model, we positioned the carotenoid to be spanning both monomers equally. Atomistic models of CCP2 dimers were validated by matching experimental and theoretical p(r) functions (Fig. 4B,C) or SAXS profiles (Figure S4 and Table S1). Additionally, the absence of a trimer (or monomer) form of holo-CCP2 from both SEC-SAXS and SEC-UV analyses indicates that the carotenoid is likely shared between two proteins and possibly prevents them from dissociating. The p(r) function of the tetrameric state of the holo-CCP2 showed a significantly more extended shape than the apo-CCP2 tetramer (Fig. 4C,E). The larger Rg together with changes in p(r) function indicates a more extended form of holo-CCP2 tetramer than the apo-CCP2 tetramer (Fig. 4C,E and Table S1). We can rationalize this with a model of holo-CCP2 tetramer that contains two holo-CCP2 dimers in an antiparallel arrangement that are interacting through a secondary CCP-CCP interface observed in the apo-CCP2 tetramer (Fig. 4E).

By combining our solution state models of CCP2 dimers together with the secondary intermolecular interface observed in the crystal structure of the apo-form we built an atomistic model of apo- and holo-tetramers. Both apo- and holo-CCP2 tetrameric models fit well with the experimental SAXS data (Fig. 4B,C and Figure S4) and suggest an asymmetrical arrangement of apo-CCP2. The asymmetry of apo-CCP2 tetramer accounts for how the apo-form is able to form a trimer in solution; this trimer cannot form from the holo- state in which the carotenoid bridges the two CCP2 domains (Fig. 4E).

### Characterization of the inter-protein S–S bond in the holo-CCP2

**Figure 5:**
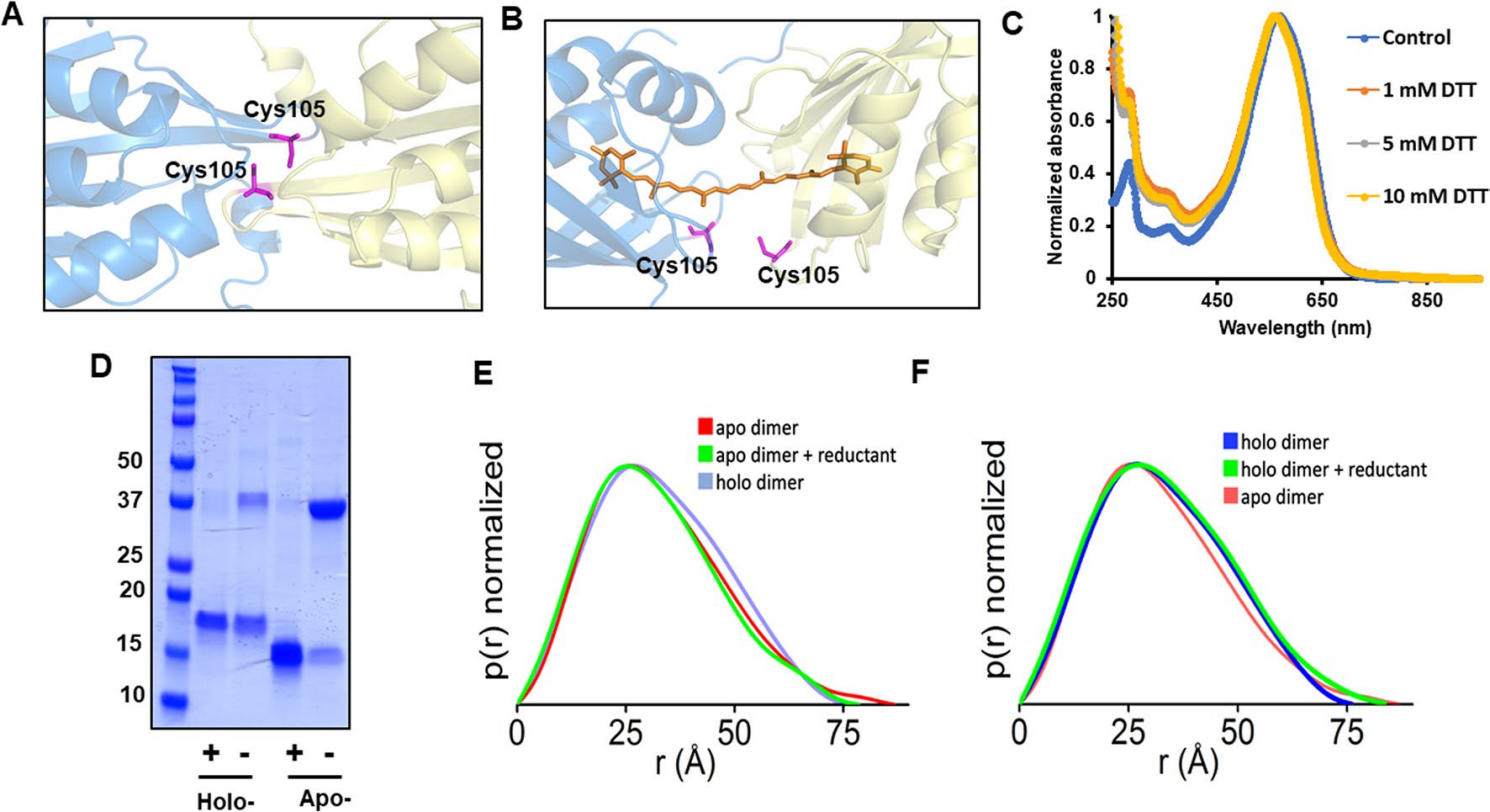
Analysis of the conserved cysteine in CCP2. Position of the cysteine in the apo- (**A**) and holo- (**B**) structural models of CCP2 dimer. (**C**) Absorption spectrum of the holo-CCP2 with different concentrations of DTT. (**D**) Analysis of the presence of the disulfide bond by SDS-PAGE with and without reductant. Numbers on the left correspond to the molecular weight marker (lane 1) in kDa. (**E**) P(r) functions obtained for the SAXS profile of apo-CCP2 measured in solution with (5 mM TCEP) (green line) and without reductant (red line) in comparison to the holo-CCP dimer (blue line). (**F**) P(r) functions obtained for the SAXS profile of holo-CCP2 measured in solution with (green line) and without reductant (blue line) in comparison to the apo-CCP dimer (red line). Experimental SAXS profiles used to calculate P(r) functions are shown in the Figure S4.

The CCP2 subtype of CCPs is defined by a single conserved cysteine at position 105 (Figure S1). We confirmed the presence of a disulfide bond in *Tolypothrix* CCP2 using denaturing gel electrophoresis (Fig. 5D). When holo- and apo-CCP2 were run on SDS-PAGE with reductant, the amount of the dimer band is substantially decreased and the monomer becomes the most prominent band, suggestive of a reducible disulfide bond in the dimer. Accordingly, in our models for apo- and holo-CCP2 dimers, we oriented the monomers to place the Cys105 residues within disulfide bonding distance (Fig. 5A,B). To probe the effect of the disulfide bond on the spectral properties, the holo-CCP2 was treated with different concentrations (1, 5, and 10 mM) of different reductants (DTT and TCEP), and the absorption spectrum was measured either immediately or after 30 min incubation (Fig. 5C and Figure S5). Reducing the disulfide bond had no effect on the absorption spectra, suggesting that there was no change in the carotenoid environment. Furthermore, adding reductant to the sample did not significantly alter the SEC-SAXS-MALS profile (Fig. 5E,F), indicating that the redox state of the disulfide does not affect carotenoid binding nor the oligomeric state of the holo-CCP2.

### Identification of specific carotenoid binding residues in the CCP2

The SEC-purified dimer and tetramer peaks (Fig. 3) of apo- and holo-CCP2 were subjected to XFMS to obtain solution-state structural identification of the carotenoid- binding residues in CCP2. After X-ray irradiation, the samples were subjected to a bottom-up liquid chromatography/mass spectrometry analysis using trypsin and GluC digestion for identification and quantification of side-chain modification. The identified peptides resulted in 88% sequence coverage. The hydroxyl radical reactivity ratio of the rate constant (Figure S6), which is a measure of both intrinsic reactivity and solvent accessibility of the modified side chains, was determined from the dose–response plots of each modified peptide (see Materials and methods and Supplementary Table 2).

**Figure 6:**
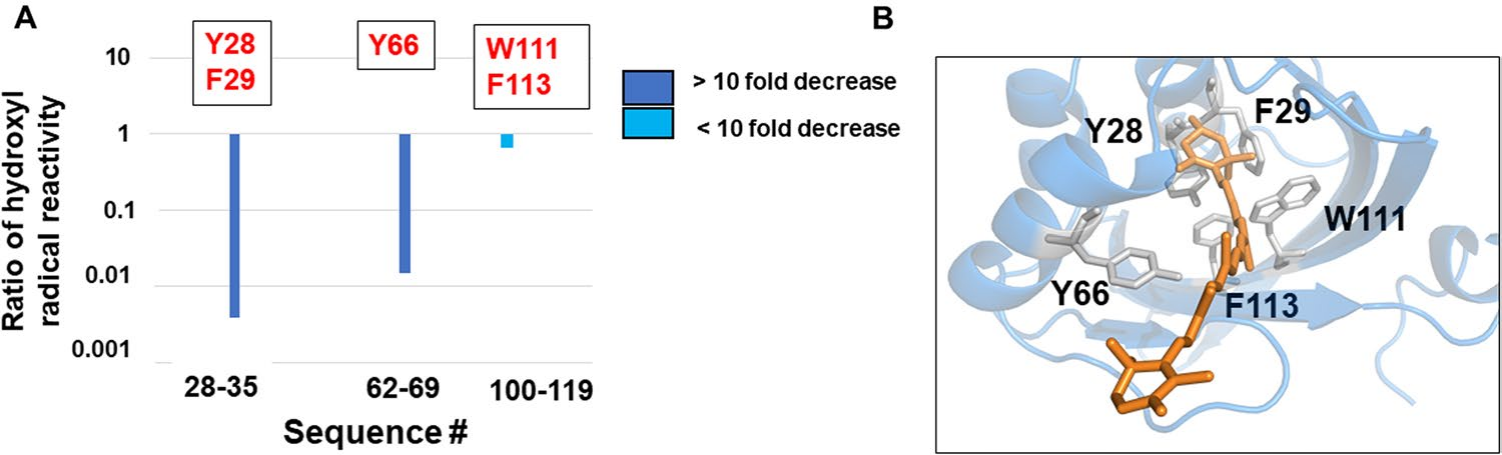
XFMS data for carotenoid-binding residues within the CCP2 dimer. (**A**) Hydroxyl radical reactivity rates for the peptides implicating carotenoid-binding residues. (**B**) The identified carotenoid-binding residues mapped onto a monomer of a holo-dimer CCP2.

The most prominent decreases in solvent accessibility when comparing apo- and holo-CCP2 are located in the binding pocket, at Y28, F29, and Y66 (> ten fold decrease in solvent accessibility). We also observed large decreases in solvent accessibility for W111 and F113 (Fig. 6A and Supplementary Table 2). It is important to highlight that residues W111 and F113 are in the same peptide with W104 (peptide 110–119). In the apo-CCP2 dimer structure (PDB 6FEJ^19^), W104 of one monomer is inserted into the central cleft of the second monomer. This intercalated arrangement is likely required in the absence of a carotenoid to shield the hydrophobic cavity within the NTF2 domain from solvent. In our holo-CCP2 dimer, W104 is more solvent-exposed than in the apo- form, in part because of the lack of intercalation between monomers but also because the two domains are slightly further apart (Figure S6). Therefore, the overall change in solvent accessibility for the peptide (110–119, 1.5-fold increase) was mitigated by reciprocal and opposite changes in accessibility of residues in the same peptide: an increase in W104 and decrease in W111 and F113 when the carotenoid is present (Fig. 6, Supplementary Table 2). All of these affected residues are highly conserved among CCPs (Figure S1), with the exception of W104 which is specifically conserved in CCP2. We detected no differences in solvent accessibility between the dimer and tetramer forms (Figure S6), consistent with the tetramer formation being unrelated to carotenoid binding.

## Discussion

In this study, we used SEC-SAXS to characterize the oligomerization states and XFMS to identify carotenoid-binding residues of the CCP2 isolated from its native host, *Tolypothrix*. We find that *Tolypothrix* CCP2 is predominantly a dimer (Fig. 3) with a single carotenoid molecule spanning the two monomers (Figs. 4A,D, 5A,B and S6). In addition, although our models of the apo- and the holo-CCP2 dimer suggest the conserved cysteine at position 105 is located at the interface between the two monomers where it forms a disulfide bond (Fig. 5A,B), the oligomerization state of CCP2 does not appear to be redox-sensitive. The disulfide may, however, play a role in some other regulatory response.

Despite the presence of a variety of carotenoids in *Tolypothrix*, including β-carotene, echinenone (ECN), and myxoxanthophyll^38^, CCP2 exclusively binds CAN. Interestingly, we observed two isomers of CAN in our CCP2 preparations from *Tolypothrix*. However, because carotenoids with long conjugated chains can spontaneously isomerize at room temperature^39^, the biological significance of these isomers is unclear. To model the carotenoid within the CCP2 we used the CTD of the OCP as a template because it was a better fit to the SAXS data than the apo-CCP2 structure. The slight separation of the two monomers required to fit CAN within the CCP2 dimer model is supported experimentally by the increase in solvent accessibility of the highly conserved residue W104 that is at the dimer interface (Figure S6), as well as the increased Rg (Supplementary Table 1). Furthermore, unlike the apo-CCP2 structure^19^, our holo-CCP2 dimer model confirmed that the C-terminal helix is displaced when the carotenoid is present leaving the cavity accessible for the pigment.

We identified with certainty a minimum of five residues directly interacting with the carotenoid: Y28, F29, Y66, W111 and F113 (Fig. 6). Notably, the absolutely conserved tyrosine (Y201) and tryptophan (W288) in the CTD of the OCP that hydrogen bond to the keto ring of the carotenoid and are essential for photoactivation^8,40^ are also absolutely conserved in the CCP2 as Y28 and W111 (Figure S1). Both were identified here as interacting with the pigment, suggesting that in CCP2 the keto-oxygens of CAN form hydrogen bonds to the sidechains of these residues in both monomers, stabilizing the dimer.

Spectroscopically, the extreme red shift of absorption maximum of CAN induced by the binding to CCP2 (~ 80 nm) is the largest reported for any OCP-related protein. For comparison, the largest known carotenoid shift (> 100 nm) is the binding of asthaxanthin to the lobster protein crustacyanin^5^. Some OCP mutants exhibit absorption maximum at around 525 nm^41^, and HCP binding CAN has a peak absorption at ~ 530 nm^9,14^. The origin of this significant red-shift is most likely due to CCP-specific protein-carotenoid interactions; Y28 and W111 likely hydrogen bond to the keto-oxygen of the CAN in CCP2. It is known that the properties of the carotenoid-binding pocket in the CTD of the OCP require that the β-ring, containing a keto-oxygen, is in the s-trans configuration in order to form hydrogen bonds between the keto-oxygen and tryptophan (W288) and tyrosine (Y201) in the binding pocket^8^. Since the lowest energy configuration of a β-ring in solution is s-cis^42^, such change in configuration leads to prolongation of effective conjugation length of the bound carotenoid and consequently a red shift of the absorption spectrum^43^. In CCP2, both β-rings of CAN reside in a CTD-like environment, indicating that both terminal rings are in the s-trans configuration, further prolonging the effective conjugation (Fig. 4D). This conclusion is also supported by recent resonance Raman data on CCP2 from *Anabaena*, showing that the frequency of the C=C stretching mode corresponds to an effective conjugation N_eff_ > 13, thus much longer than for OCP^44^.

It must be noted, however, that switching both β-rings to s-trans configuration cannot solely explain the observed red shift in CCP2. If we use a ‘reference’ carotenoid, rhodoxanthin, which belongs to a family of retro-carotenoids and therefore has both β-rings in the s-trans configuration even in solution, its absorption spectrum peaks at 505 nm in benzene^45^. Thus, the prolongation of effective conjugation due to the twist of β-rings to s-trans configuration accounts only for about one third (~ 1,000 cm^−1^) of the observed red shift. The rest of the red-shift in CCP2 must be related to CAN-protein interactions, though hydrogen bonding of the keto-oxygen to tryptophan/tyrosine may also contribute. Recently, Bondanza et al. used multiscale atomistic modelling to successfully reproduce absorption spectra of OCP and RCP. They showed that electrostatic effects caused by charged amino acids are the primary cause of the red shift of the RCP absorption spectrum^46^. The same effects likely contribute to the extreme red shift of CAN in CCP2, though it is impossible to quantify the possible role of charged amino acids without a detailed atomic structure of CCP2.

A further shift of the absorption spectrum is observed in the tetramer (Fig. 2B) that is formed at high concentrations of holo-CCP2 (Figure S3B). In the tetramer, two CAN molecules are arranged as a head-to-tail dimer (β2-ring of CAN in the first CCP2 is at the level of the β1 ring of CAN in the second CCP2), indicating that the further shift of absorption maximum from dimer to tetramer (Fig. 2B) could be caused by an excitonic interaction between the two CANs in the tetramer. A comprehensive study of the changes in the absorption spectra of carotenoid dimers were reported for a similar ketocarotenoid astaxanthin^47^. This study showed that for a head-to-tail dimer with 4 Å distance between the carotenoids the red-shift can reach 1,000 cm^−1^. However, the distance between the β2 and β1 rings of the two CANs in the tetramer is nearly 30 Å (Fig. 4E) and since the coupling strength decreases with *R*^−3^, the expected red-shift induced by the possible excitonic interaction between the two CANs should not exceed 10 cm^−1^. This is far less than the ~ 190 cm^−1^ corresponding to the observed shift from 560 to 570 nm. Instead, small changes in CAN-protein interaction upon forming the tetramer must be responsible for this shift, underscoring the importance of CAN-protein interaction in CCP2.

In addition to the extreme red shift, another interesting feature of the CCP2 absorption spectrum, as noted above, is the absence of any vibrational structure. Our data shows that the keto-oxygens of CAN are hydrogen bonded to the side chains of W111 and Y28 in both monomers, stabilizing the dimer; therefore, the CAN should be locked in the binding pocket and, consequently, the conformational disorder should be significantly restrained. Such conditions should increase the resolution of vibrational bands in the absorption spectrum, which is not observed. Interestingly, *Anabaena* CCP2 with CAN behaved as a dimer; in a CCP2 binding ECN, the dimer was unstable^17^. CAN, in contrast to ECN, is symmetrical with keto-oxygens on both terminal rings; Slominsky et al. have stressed the importance of the coordinating residues forming the hydrogen bonds between the keto-groups of CAN^17^. Locking the carotenoid in the binding pocket by the H-bonds to W288 and Y201 is the reason for better resolution of vibrational bands in OCP compared to solution. Indeed, W288A/Y201A OCP double mutant, has less-resolved vibrational bands due to the inability to form H-bonds with the carotenoid keto-oxygen in the CTD^44^. Recent molecular dynamics data clearly showed that loss of vibrational structure in the absorption spectrum is related to larger conformational mobility of the carotenoid^46^, suggesting that CAN in CCP2 must have some conformational freedom. The possible explanation of this seemingly contradictory situation could be inferred from the holo-CCP2 model shown in Figs. 4D, 5A or 6B. Since the two CCPs are not connected and there is a gap between the two domains, the presumed conformational freedom may be caused by rotation of the whole domain, with CAN as the rotation axis. Then, even though the β-rings are restrained in the CCP2 binding pocket by H-bonds to tyrosine and tryptophan, there is still a conformational landscape of CAN caused by variation in the mutual orientation of the two domains.

In conclusion, our structure of the CCP2 explains how the carotenoid is allocated within the dimer and provides the first insight into how the protein environment establishes the unusual spectral properties of the bound carotenoid. Such insights provide another step toward understanding the role of the CCP, for which the biological function remains enigmatic. Furthermore, the CCP2 is a member of the widespread NTF2-like superfamily and our results unequivocally establish that this structural fold, with its hydrophobic internal cavity, is also capable of being a carotenoid-binding domain. Given the ubiquity of this domain across all kingdoms of life, it may be that other organisms, such as photosynthetic algae and plants have members of this carotenoprotein family that await to be discovered.

## Supplementary information

Supplementary Information.
