# Supplementary material for: Structural analysis of a new carotenoid-binding protein: the C-terminal domain homolog of the OCP
Source: Sci Rep. 2020 Sep 23;10:15564. doi: 10.1038/s41598-020-72383-y (PMC7512017; doi:10.1038/s41598-020-72383-y)
Supplement: Supplementary file 1 — Supplementary Information. [file 41598_2020_72383_MOESM1_ESM.docx]

**Supplementary Information**

**Structural analysis of a new carotenoid-binding protein: the C-terminal domain homolog of the OCP**

Maria Agustina Dominguez-Martin^1,2^, Michal Hammel^5^, Sayan Gupta^5^, Sigal Lechno-Yossef^2^, Markus Sutter^1,2^, Daniel J. Rosenberg^5,6^, Yan Chen^7^, Christopher J. Petzold^7^, Corie Y. Ralston^8^, Tomáš Polívka^4^ & Cheryl A. Kerfeld^*1,2,3^

^1^ Environmental Genomics and Systems Biology and Molecular Biophysics and Integrated Bioimaging Divisions, Lawrence Berkeley National Laboratory, Berkeley, CA 94720, USA

^2^ MSU-DOE Plant Research Laboratory, Michigan State University, East Lansing, MI 48824, USA

^3^ Department of Biochemistry and Molecular Biology, Michigan State University, East Lansing, MI 48824, USA

^4^ Institute of Physics, Faculty of Science, University of South Bohemia, Branišovská 31, 370 05 České Budějovice, Czech Republic

^5^ Molecular Biophysics and Integrated Bioimaging Division and Molecular Foundry, Lawrence Berkeley National Laboratory, Berkeley, CA 94720, USA

^6^ Graduate Group in Biophysics, University of California, Berkeley, CA 94720, USA

^7^ Biological Systems and Engineering Division, Lawrence Berkeley National Laboratory, Berkeley, CA 94720, USA

^8^ Molecular Foundry, Lawrence Berkeley National Laboratory, Berkeley, CA 94720, USA

* Address correspondence to: [ckerfeld@lbl.gov](mailto:ckerfeld@lbl.gov)

The author responsible for distribution of materials integral to the findings presented in this article is: Cheryl A. Kerfeld ([ckerfeld@lbl.gov)](mailto:ckerfeld@lbl.gov)).

Address: MSU-Department of Energy Plant Research Laboratory, Michigan State University, East Lansing, Michigan 48824, USA

**Supplementary Material and Methods:**

**Phylogenetic analysis**

A total of 225 unique sequences of full length OCP (136 sequences) and CCP (89 sequences were retrieved from Integrated Microbial Genomes database (IMG; <https://img.jgi.doe.gov/>) in a similar manner to that described by (Kerfeld et al., 2017)^1^. The sequences were aligned using MUSCLE ^2^, the alignments were trimmed manually to retain only the CTD domain of the OCP, removing NTD and the flexible linker, and re-aligned. The multiple sequence alignment was used to construct a phylogenetic maximum likelihood tree, using RaxML ^3^. From the phylogenetic analysis, as well as from information about the identity of the residue corresponding to F278 in *Synechocystis* sp. PCC 6803 OCP1 (residue C105 in *Fremyella* CCP2), which is either cysteine in CCP2 or phenylalanine in CCP1 and OCP1, sequences were grouped together and re-aligned. Multiple sequence alignments were submitted at the skylign website (<http://skylign.org/>) to construct HMM logos using observed counts of amino acids in each alignment position.

**Supplementary Figures and Tables:**

Supplementary Table 1: MALS and SAXS parameters

| Sample | SEC-MALS MW (kDA) | SAXS MW (kDA) | Rg from Guinier plot  (Å) | Rg from p(r) function | Maximal dimension Dmax  (Å) | SAXS model fit Χ^2^ |
| --- | --- | --- | --- | --- | --- | --- |
| apo CCP2 dimer | **35-45** | **~36** | **24.0±0.4** | **24.3±0.8** | **~80** | **1.1** |
| apo CCP2 trimer | **65** | **~60** | **30.2±0.8** | **30.8±0.9** | **~115** | **1.1** |
| apo CCP2 tetramer | **80-100** | **~72** | **31.5±0.5** | **31.9±0.7** | **~115** | **1.1** |
| holo CCP2 dimer | **40-50** | **~36** | **25.5±0.8** | **26.5±0.8** | **~80** | **1.7** |
| holo CCP2 tetramer | **70-80** | **~86** | **32.5±0.4** | **33.3±0.9** | **~120** | **2.6** |

**
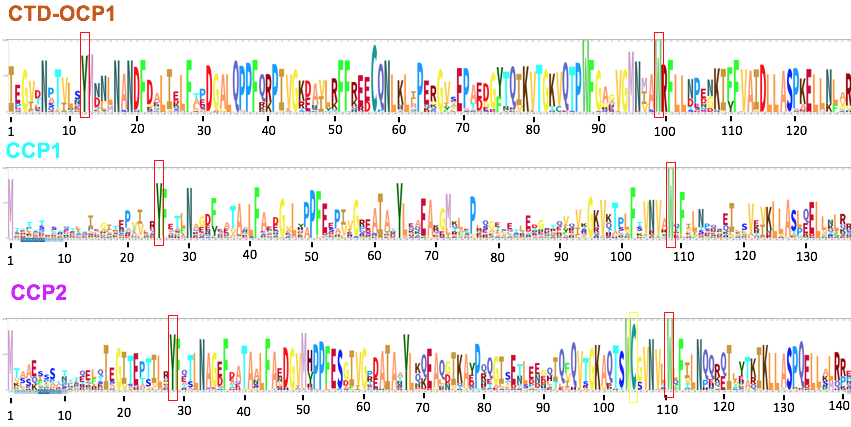
**

**Figure S1: Sequence conservation among CCPs evolutionary subtypes.** HMM logos are shown for the OCP1-CTD and CCPs subfamilies of the pfam02136 domain. The red boxes highlight the conserved tryptophan and tyrosine and the yellow box highlights the conserved cysteine which characterizes CCP2 subtype.

**
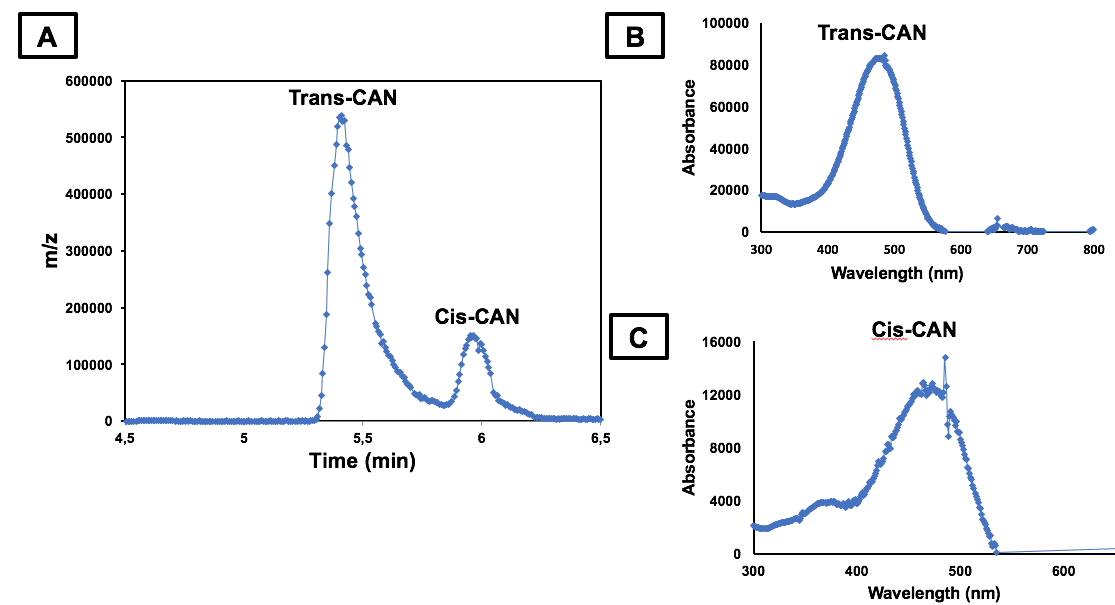
**

**Figure S2: Detection of cis- and trans- isomers of canthaxanthin.** A. LC-DAD-MS chromatograms of CAN in CCP2 sample. B, C. Absorbance spectrum of each peak.

**
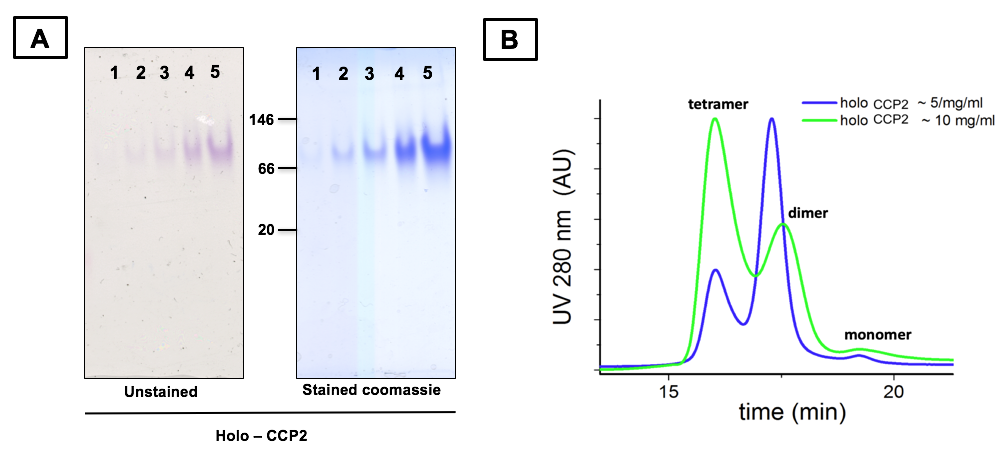
**

**Figure S3: A. Native-PAGE for holo-CCP2.** Different protein concentrations were loaded in each gel lane: 1 = 0.25 mg/mL; 2 = 0.5 mg/mL; 3 = 0.75 mg/mL; 4 = 1.85 mg/mL and 5 = 2.6 mg/mL. Unstained and Coomassie blue-stained native gel. **B.** **Chromatogram for holo-CCP2 at different concentrations.** UV 280 nm profile for 5 mg/mL holo-CCP2 (blue line) and for 10 mg/mL holo-CCP2 (green line). The elution peaks for the tetramer, dimer, and monomer forms are labeled.

**
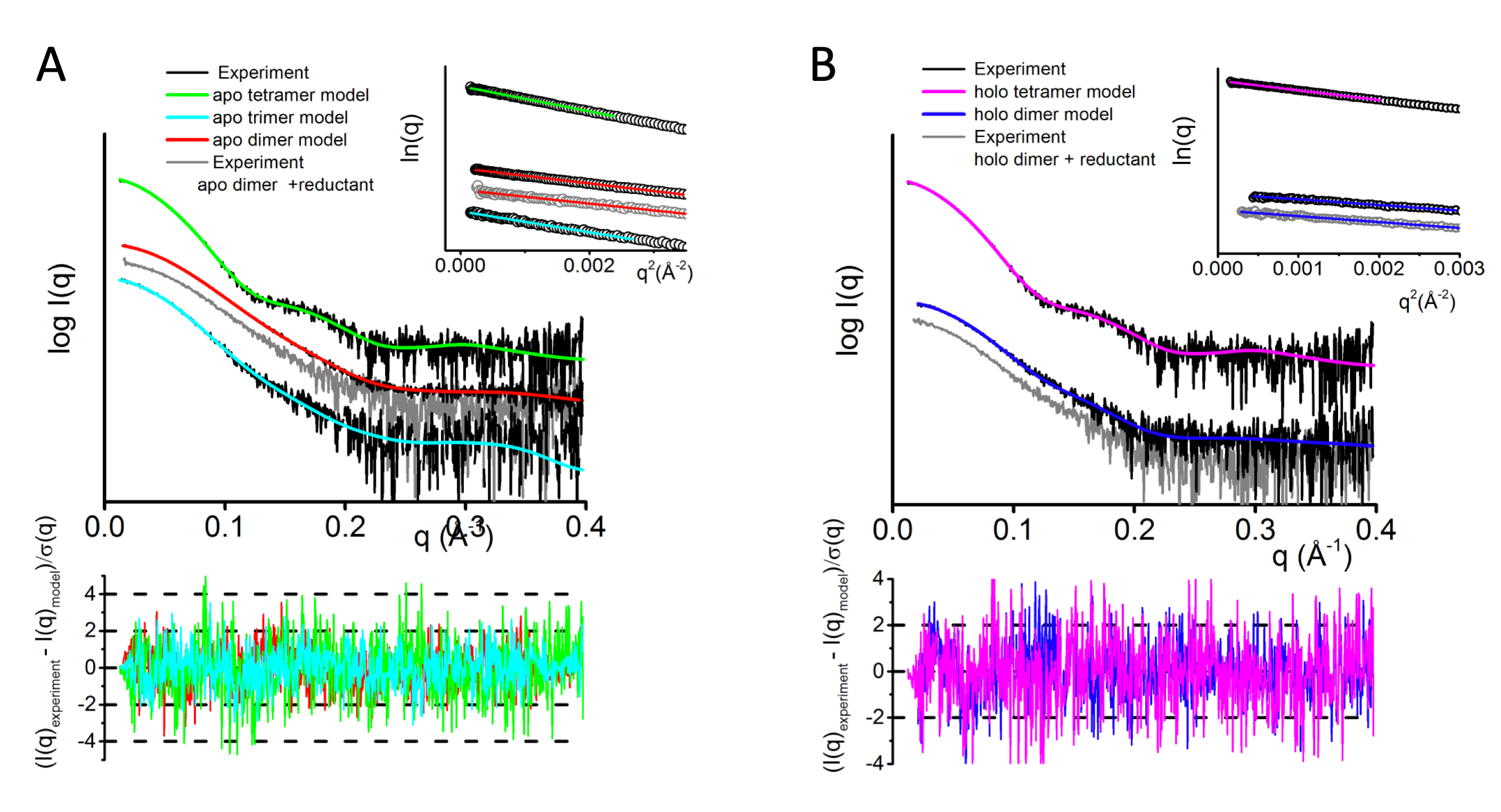
**

**Figure S4: In depth SAXS data.** A-B) Experimental SAXS profiles (black) of CCP2 in the absence (A) or presence (B) of bound carotenoid, displayed with the calculated scattering from the atomistic models shown in Figure 4, together with fit-residuals. Experimental SAXS profile (gray) of CCP2 dimer in the presence of reductant. Inset shows the Guinier plot of experimental SAXS profiles with the linear fit in the q×Rg < 1.5 limits.

**
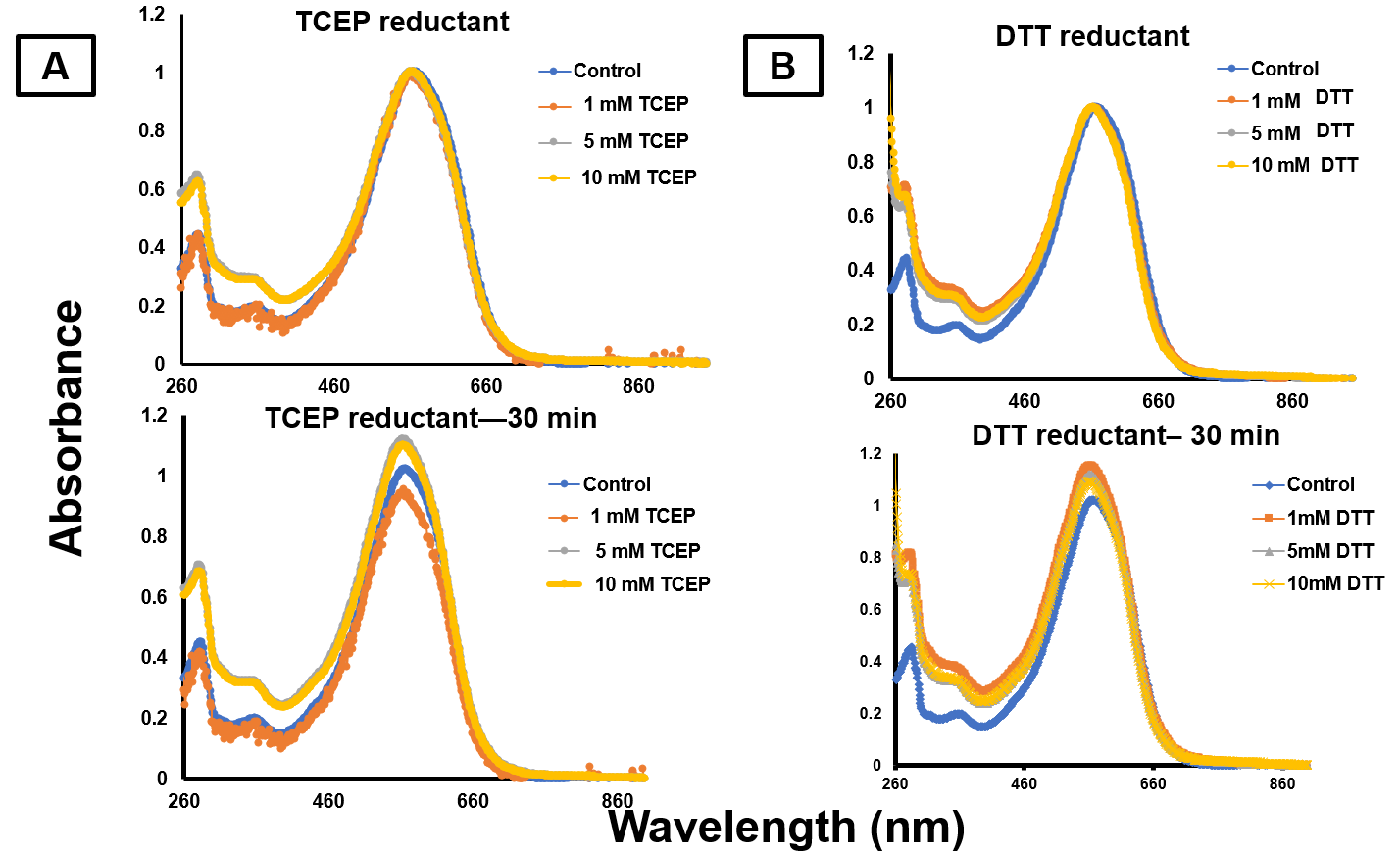
**

**Figure S5: Absorption spectra of CCP2. A.** Different concentrations of TCEP were added to the CCP2 samples and the absorption spectra were measured without incubation and after 30 min of incubation (above and below, respectively). **B.** Different concentrations of DTT were added to the CCP2 samples and the absorption spectra were measured without incubation and after 30 min of incubation.


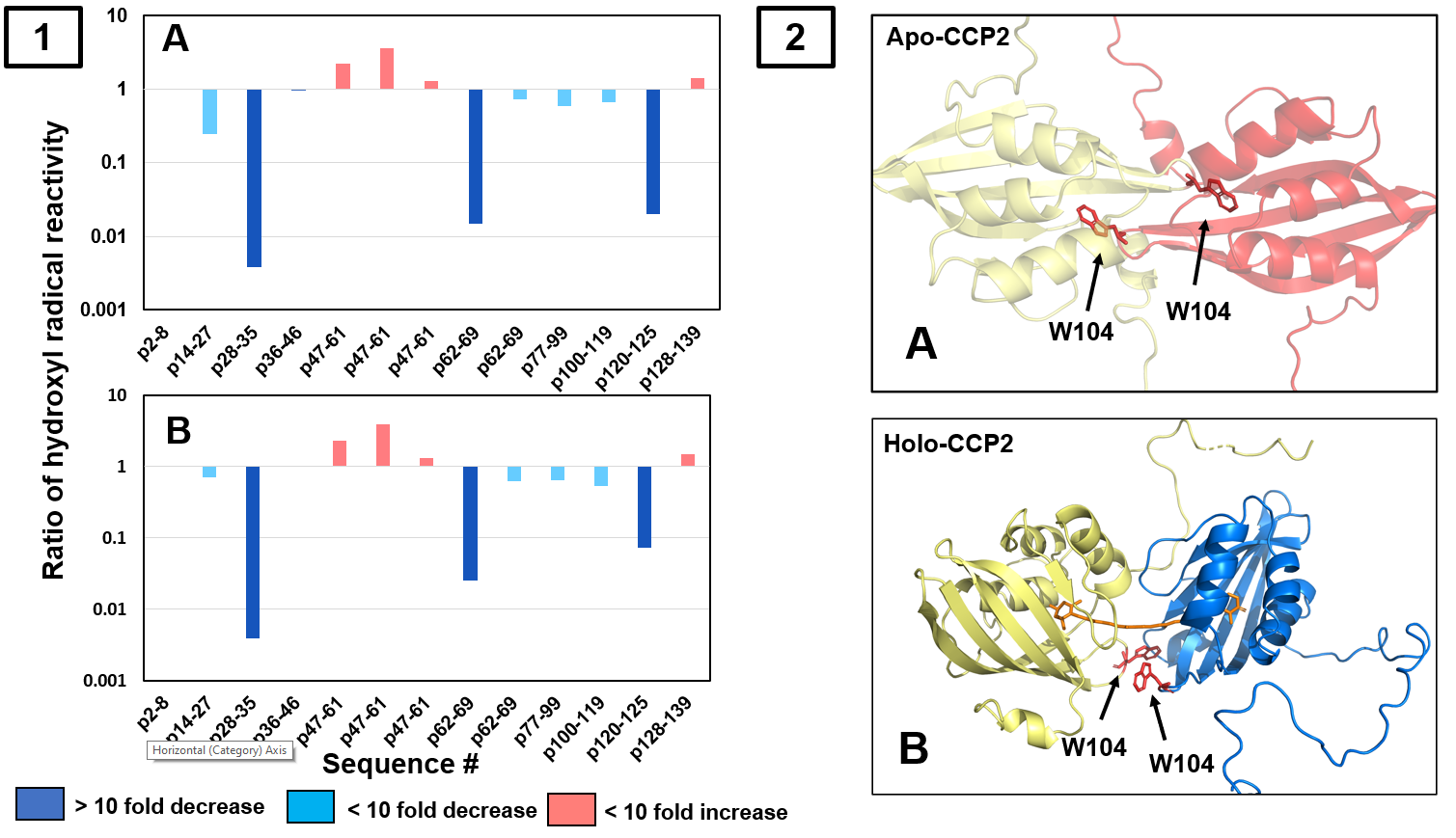


**Figure S6: 1. Ratio of hydroxyl radical reactivity during X-ray footprinting.** Ratios are shown, as described in Supplementary Table 2, for each peptide comparing holo-dimer with apo-dimer CCP2 (A) and holo-tetramer with apo-tetramer CCP (B). **2.** **Position of the conserved W104.** The position of the conserved W104 residue is indicated ono the modelled structures for the apo-CCP2 (A) and in the holo-CCP2 (B) dimers.


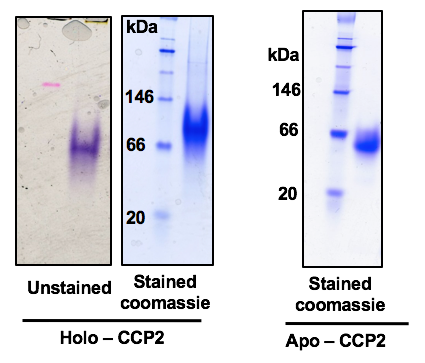


**Figure S7:** Native gel for holo-CCP2 (left, unstained and coomassie blue-stained) and apo-CCP2 (right).


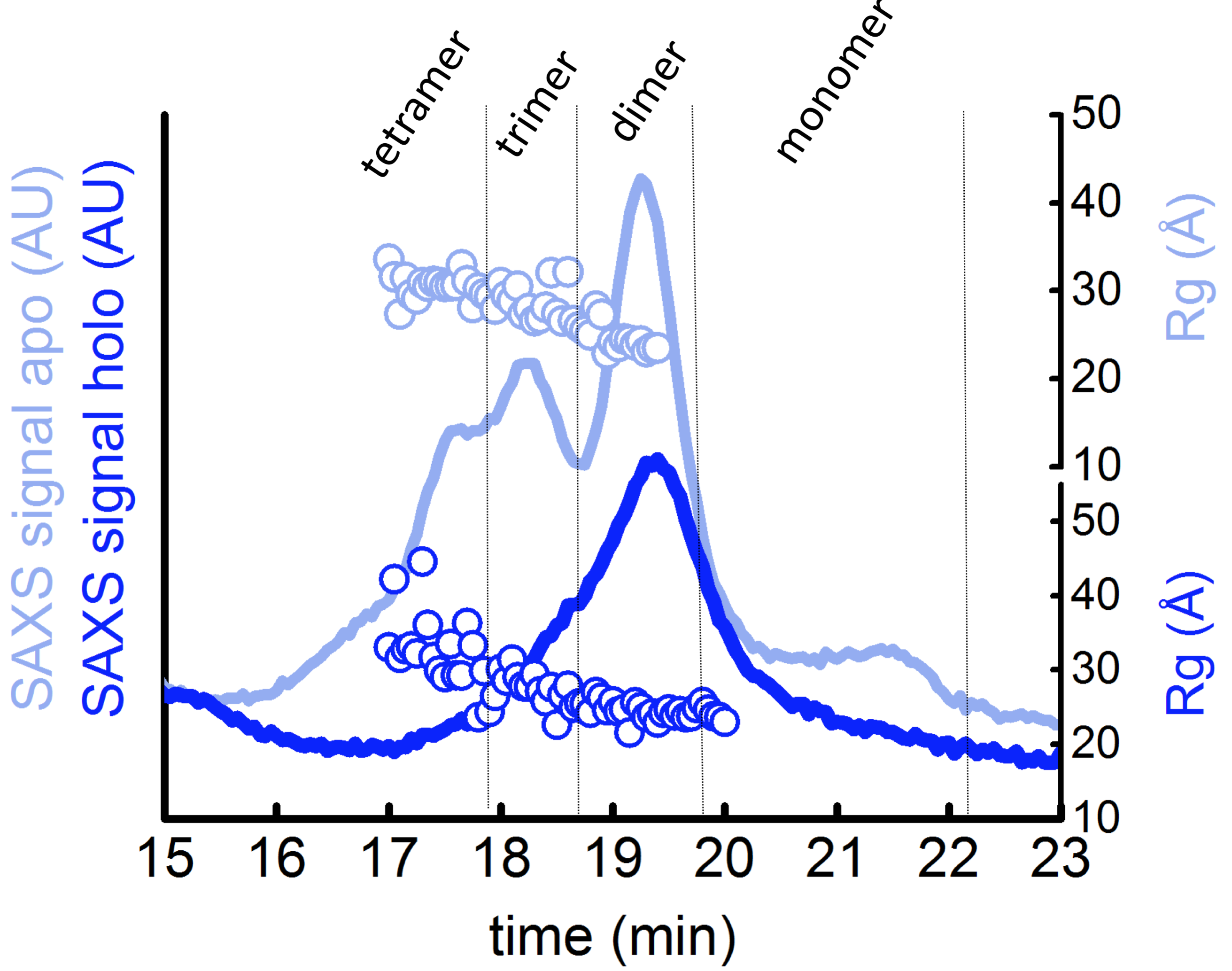


**Figure S8:** SEC-SAXS chromatograms. Chromatograms are shown for apo-CCP2 (light blue) and holo-CCP2 (dark blue). Solid lines represent the integrated SAXS signal in arbitrary units, while symbols represent Rg values for each collected SAXS frame versus elution time.

**
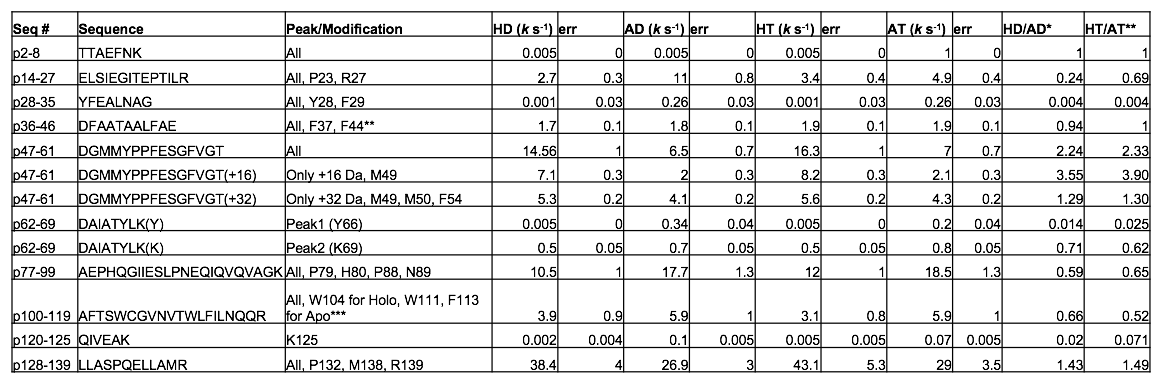
**

**Supplementary Table 2: The ratio of hydroxyl radical reactivity. Seq #** = sequence number of the residues; **err** = error value. **HD** = Holo-dimer; **AD** = Apo-dimer; **HT** = Holo-tetramer; **AT** = Apo-tetramer.  **(*)** Ratio between apo-dimer and holo-dimer. **(**)** Ratio between apo-tetramer and holo-tetramer. **(***)** Peptide with reciprocal changes observed.

**
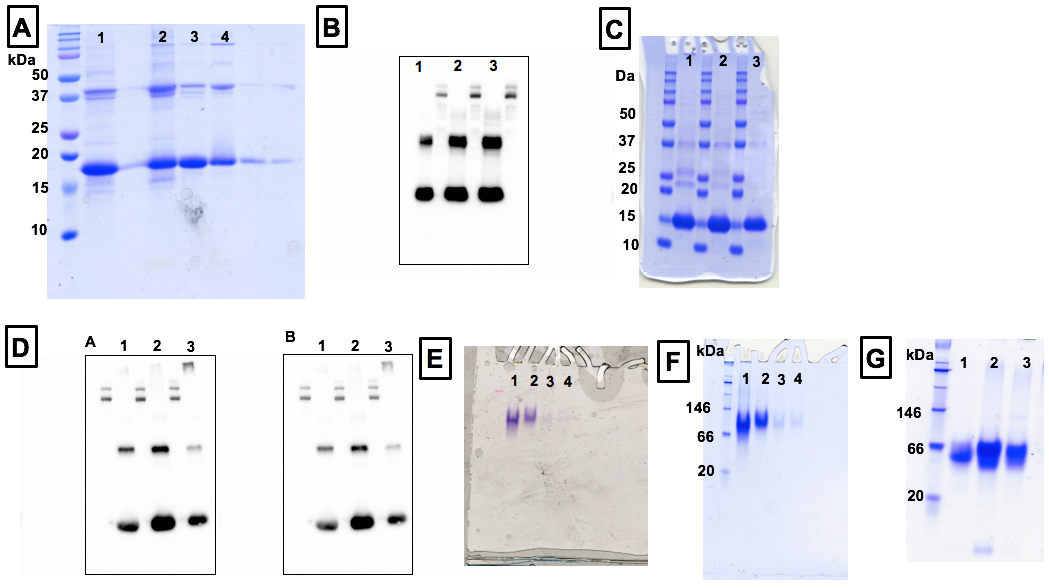
Supplementary Figure: A. Full length gel from Figure 1 Holo-CCP2 SDS-PAGE.** Lanes 1-4 different fractions from the purification. **B. Full length anti-His immunodetection from Figure 1 Holo-CCP2 blotting.** Lanes 1-3 different volumes of holo-CCP2 samples. **C. Full length gel from Figure 1 Apo-CCP2 SDS-PAGE.** Lanes 1-3 different volumes of apo-CCP2 samples. **D. Full length anti-His immunodetection from Figure 1 Apo-CCP2 blotting (A and B different exposition times).** Lanes 1-3 different volumes of apo-CCP2 samples. **E. and F. Full length unstained and coomassie-blue Native gel for holo-CCP2 from Figure S7.** Lanes 1-4 different volumes of holo-CCP2 samples. **G. Full length coomassie-blue Native gel for apo-CCP2 from Figure S7.** Lanes 1-3 different volumes of apo-CCP2 samples.

**References**

1 Kerfeld, C. A., Melnicki, M. R., Sutter, M. & Dominguez-Martin, M. A. Structure, function and evolution of the cyanobacterial orange carotenoid protein and its homologs. *New Phytol* **215**, 937-951, doi:10.1111/nph.14670 (2017).

2 Edgar, R. C. MUSCLE: a multiple sequence alignment method with reduced time and space complexity. *BMC Bioinformatics* **5**, 113, doi:10.1186/1471-2105-5-113 (2004).

3 Stamatakis, A. RAxML version 8: a tool for phylogenetic analysis and post-analysis of large phylogenies. *Bioinformatics* **30**, 1312-1313, doi:10.1093/bioinformatics/btu033 (2014).
